# Supplementary material for: Adsorption of Hexavalent Chromium Using Activated Carbon Produced from Sargassum ssp.: Comparison between Lab Experiments and Molecular Dynamics Simulations
Source: Molecules. 2022 Sep 16;27(18):6040. doi: 10.3390/molecules27186040 (PMC9503432; doi:10.3390/molecules27186040)

Supplementary Materials

Adsorption of hexavalent chromium using activated carbon produced from *Sargassum spp*: Comparison between lab experiments and molecular dynamic simulations

Yeray Alvarez-Galvan[1,2], Babak Minofar[3*], Zdeněk Futera[4], Marckens Francoeur[1], Corine Jean-Marius[1], Nicolas Brehm[2], C. Yacou[1], Ulises J. Jauregui-Haza[5], S. Gaspard[1*]

[1] Laboratoire COVACHIM-M2E, Université des Antilles, Campus de Fouillole, 97157, Pointe à Pitre Cedex, Guadeloupe.

[2] NBC SARL Company, 8, Rue Saint Cyr, résidence Océane - Apt n°5 – 97300, Cayenne

[3] Laboratory of Structural Biology and Bioinformatics, Institute of Microbiology of the Czech Academy of Sciences, Zamek 136, 37333 Nové Hrady, Czech Republic

[4] Faculty of Science University of South Bohemia in České Budějovice Branišovská 1760/31a, 37005, České Budějovice, Czech Republic

[5] Instituto Tecnológico de Santo Domingo (INTEC), Dominican Republic

[*]***Corresponding authors:*** Sarra Gaspard, Babak Minofar

Laboratoire COVACHIM-M2E, Université des Antilles, 97157, Pointe à Pitre Cedex, Guadeloupe.

Email address: sarra.Gaspard@univ-antilles.fr; minofar@nh.cas.cz

Tel.: +590 690 55 47 89

Table of Contents

Table S 1. Atomic type parameters: atomic masses [AMU] and Lennard-Jones-potential parameters R [Å] and ε [kcal/mol]. **3**

Table S 2. Bond type parameters: harmonic-potential force constant k_B [kcal/mol/Å^2] and equilibrium distance r_0 [Å]. **4**

Table S 3. Valence-angle type parameters: harmonic-potential force constant k_A [kcal/mol/deg^2] and equilibrium angle value φ_0 [deg]. **4**

Table S 4. Dihedral-angle type parameters: force constant k_D [kcal/mol], equilibrium angle value ϕ_0 [deg] and periodicity n (negative value denotes multi-term potential form). **4**

Table S 5. Comparison of vibrational frequencies [cm-1] of vacuum Cr^{VI} anions as calculated in DFT (PBE0/Aug-CC-pVDZ) and in force field (FF) using the fitted parameters. The frequencies were calculated by the harmonic-approximation approach from the second derivatives of the total energy with respect to atomic coordinates. **4**

Figure S 1. Atom types (red symbols), atomic point charges (black numbers, atomic units) and indicated inter-atomic bonding (black lines) in (a) CrO42-, (b) HCrO4-, and (c) Cr2O72- anions. **5**

Figure S 2. Plot of ln(Keq) vs 1/K. **5**

Force field parameters for the Cr^{VI} anions

In order to simulate the interaction of activated carbon model with Cr^{VI} ions, force field parameters have been derived. The molecular-mechanical models for Cr^{VI} ions were described by the following potential function based on general Amber force field (GAFF) parameters [1], [2].

$$V(r) = \sum_{bonds} k_B(r - r_0)^2 + \sum_{angles} k_A(\varphi - \varphi_0)^2 + \sum_{dihedrals} \frac{k_D}{2}[1 + \cos(n\phi - \phi_0)] + \sum_{pairs} \varepsilon_{ij}\left[\left(\frac{R_{ij}}{r_{ij}}\right)^{12} - 2\left(\frac{R_{ij}}{r_{ij}}\right)^{6}\right] + \frac{1}{4\pi\varepsilon_0}\sum_{pairs}\frac{q_i q_j}{r_{ij}}$$

Atom-type assignment of Cr(VI) species and their inter-atomic bonding are specified in the supplementary Figure S 1 together with the values of the atomic charges q_i. The charges were fitted by RESP method [3] from the electrostatic potential evaluated by density functional theory (DFT) at PBE0 [4] /Aug-CC-pVDZ [5], [6] level on the optimized ionic structures by using Gaussian 16 package [7]. For the activated carbon molecule, the B3LYP/6-31G* was used. For the Lennard-Jones-potential parameters R and ε were taken from the General Amber Force Field (GAFF) [1], [2] and applied without any modifications, the binding descriptors k_B, k_A, k_D, r_0, φ_0, and ϕ_0 were further refined in order to reproduce DFT vibrational frequencies of the considered Cr^{VI} ions in the vacuum. The final binding parameters are listed in the supplementary Table S 1, Table S 2, Table S 3, and Table S 4, while the obtained vibrational frequencies are collected in supplementary Table S 5.

Table S 1. Atomic type parameters: atomic masses [AMU] and Lennard-Jones-potential parameters R [Å] and ε [kcal/mol].

Type	Mass	R	ε
ho	1.0008	0.0000	0.0000
oc	15.9999	1.6612	0.2100
od	15.9999	1.7210	0.2104
oe	15.9999	1.6837	0.1700
cr	51.9961	1.3440	0.0085

Table S 2. Bond type parameters: harmonic-potential force constant k_B [kcal/mol/Å^2] and equilibrium distance r_0 [Å].

Bond	k_B	r_0
od-ho	550.9524	0.9754
cr-oc	324.4167	1.6795
cr-od	231.5857	1.8497
cr-oe	260.1321	1.7946

Table S 3. Valence-angle type parameters: harmonic-potential force constant k_A [kcal/mol/deg^2] and equilibrium angle value φ_0 [deg].

Angle	k_A	φ_0
oc-cr-oc	70.8771	109.471
oc-cr-od	70.8771	109.471
oc-cr-oe	70.8771	109.471
cr-oe-cr	46.7234	154.830
cr-od-ho	34.7476	107.065

Table S 4. Dihedral-angle type parameters: force constant k_D [kcal/mol], equilibrium angle value ϕ_0 [deg] and periodicity n (negative value denotes multi-term potential form).

Dihedral	k_D	ϕ_0	n
oc-cr-od-ho	2.3	180.0	-2
oc-cr-od-ho	1.9	0.0	1
oc-cr-oe-cr	1.0	180.0	3

Table S 5. Comparison of vibrational frequencies [cm^{-1}] of vacuum Cr^{VI} anions as calculated in DFT (PBE0/Aug-CC-pVDZ) and in force field (FF) using the fitted parameters. The frequencies were calculated by the harmonic-approximation approach from the second derivatives of the total energy with respect to atomic coordinates.

Mode	CrO_4^{2-}		$HCrO_4^-$		$Cr_2O_7^{2-}$	
	DFT	FF	DFT	FF	DFT	FF
1	316.5	333.3	67.1	0.0	15.4	0.0
2	316.6	333.3	251.1	290.0	32.7	0.0
3	356.5	336.8	291.7	298.6	39.2	71.7
4	356.5	336.9	329.5	300.4	205.0	200.2
5	356.7	337.1	356.4	323.6	206.3	212.7

6	807.7	691.5	363.9	341.6	210.5	215.0
7	834.0	834.0	623.0	618.8	303.4	300.0
8	834.0	834.1	769.3	748.7	321.4	316.9
9	834.2	834.1	911.8	832.6	358.9	320.6
10			956.7	833.2	358.9	321.4
11			977.3	977.4	361.5	322.3
12			3702.7	3702.7	365.3	327.3
13					366.3	391.8
14					476.6	517.7
15					812.4	714.4
16					883.8	743.9
17					906.0	830.2
18					926.3	831.6
19					931.2	832.2
20					932.2	837.4
21					938.4	938.0

Figure S 1. Atom types (red symbols), atomic point charges (black numbers, atomic units) and indicated inter-atomic bonding (black lines) in (a) CrO_4^{2-}, (b) $HCrO_4^-$, and (c) $Cr2O_7^{2-}$ anions.

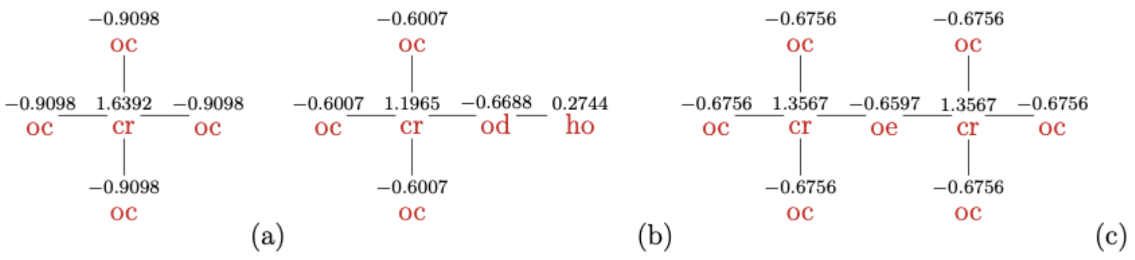

Figure S 2. Plot of ln(Keq) vs 1/K.

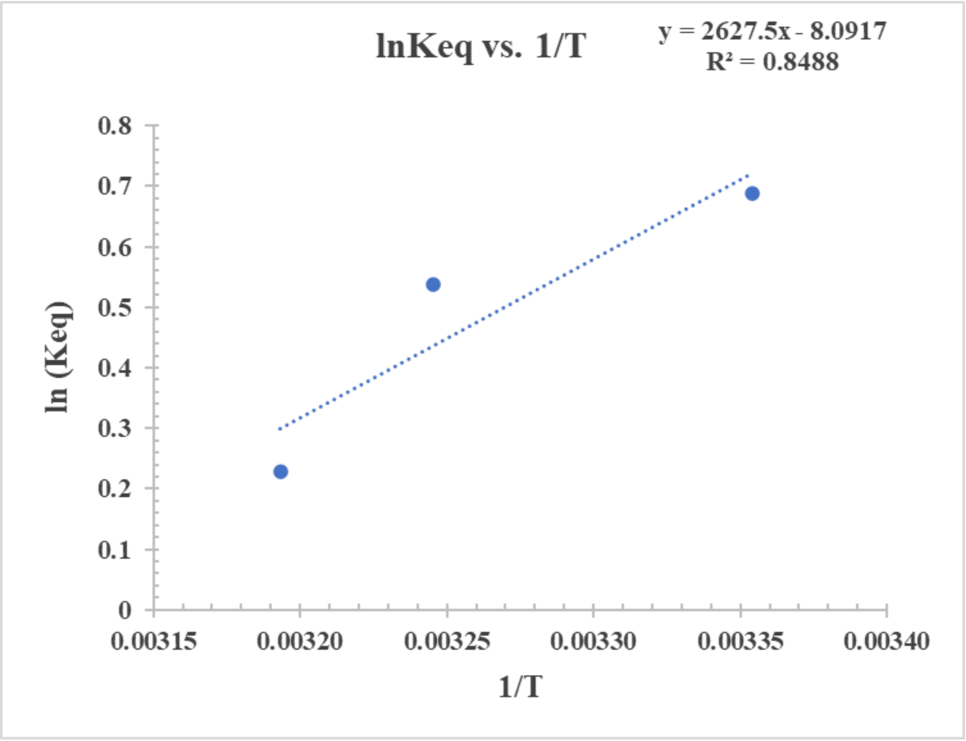

Supplement: Supplementary file 1 [file molecules-27-06040-s001.zip › molecules-1893051-supplementary.pdf]
