# Supplementary figures and images for: Adsorption of Hexavalent Chromium Using Activated Carbon Produced from Sargassum ssp.: Comparison between Lab Experiments and Molecular Dynamics Simulations
Source: Molecules. 2022 Sep 16;27(18):6040. doi: 10.3390/molecules27186040 (PMC9503432; doi:10.3390/molecules27186040)

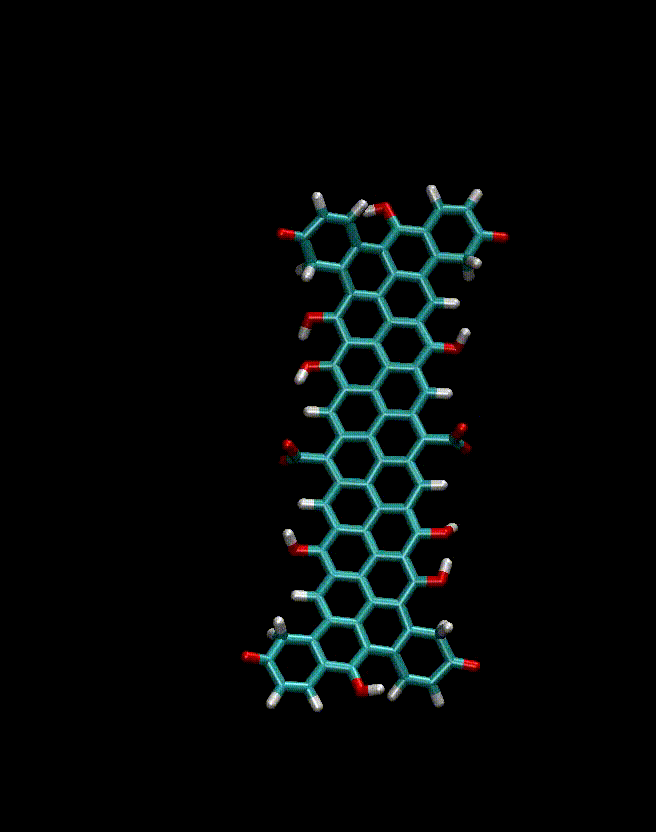

Supplement: Supplementary file 1 [file molecules-27-06040-s001.zip › molecules-1893051-supplementary.gif]
